# Clinical Analysis of the Treatment of Primary Trigeminal Neuralgia by Percutaneous Balloon Compression

**DOI:** 10.3389/fsurg.2022.843982

**Published:** 2022-02-14

**Authors:** Hui Wang, Chunhui Chen, Da Chen, Fengsheng Li, Shan Hu, Wenqian Ding, Jun Wang, Wanghua Chen

**Affiliations:** Department of Neurosurgery, Pingkuang General Hospital, Pingxiang, China

**Keywords:** trigeminal neuralgia, percutaneous balloon compression, pain, clinical effect, minimally invasive surgical

## Abstract

**Purpose:**

To summarize the technical points and clinical effects of percutaneous balloon compression (PBC) in the treatment of primary trigeminal neuralgia.

**Methods:**

The clinical data of 13 patients with trigeminal neuralgia who received PBC from April 2020 to July 2021 were retrospectively analyzed. VAS, VRS-4 and PPI were used to evaluate the postoperative pain relief. Different postoperative complications were analyzed.

**Results:**

All patients had a smooth operation, the inflation volume of the balloon was 0.7 ml, the average compression time was 120 s, and there was no balloon rupture during the operation. On the day after operation, 12 patients (92.3%) had complete pain relief, and 1 patient (7.7%) was not satisfied with pain relief, but the pain disappeared 2 weeks after the operation. After operation, there were 12 patients with facial numbness in the affected side (92.3%), 3 patients with masseter muscle weakness (23.0%), 1 patient with herpes around the mouth (7.6%), and 1 patient with diplopia (7.6%).

**Conclusion:**

PBC is an effective minimally invasive surgical method for the treatment of primary trigeminal neuralgia. It is suitable for the elderly and infirm people, those who cannot tolerate general anesthesia or are afraid of surgery, and patients who had undergone surgery but relapsed after surgery. However, it is necessary to pay attention to the serious facial numbness and postoperative masticatory weakness. These discomforts are generally relieved after half a year.

## Introduction

Trigeminal neuralgia is the most common facial neuralgia in clinic. The clinical onset is mainly unilateral pain. Most of the initial pain is a short-term phenomenon, which only appears for a few seconds or minutes. After the initial pain, it may be accompanied by a period of painless remission, and then it will attack again, with typical paroxysmal pain, severe pain, and discharge-like pain (1, 2). Under normal circumstances, the clinical manifestations of patients with trigeminal neuralgia are not obvious between the two attacks, but with the development of the disease, dull pain or even persistent pain may occur in the later stage of the disease, and the pain may last for more than 1 h. Investigations have shown that the prevalence of trigeminal neuralgia in the population ranges from 4/100,000 to 27/100,000, and the most common age is over 50 years old (3). The pathogenesis of primary trigeminal neuralgia has not been uniformly recognized by the medical community, but at present, most scholars believe that it is related to the central lesion theory and the peripheral pathogen theory. Trigeminal neuralgia not only makes the patient's language dysfunction and eating difficulty, but also may cause the patient to have bad psychology such as anxiety, irritability and depression, which has a some negative impact on the patient's daily life (4). Therefore, it is necessary to take active measures to solve trigeminal neuralgia clinically. When the trigeminal neuralgia is diagnosed for the first time, drug therapy is the first choice. Carbamazepine, as the first-line drug at present, can effectively relieve the pain. However, after taking it for a long time, the curative effect will decrease, and the patient finally stops taking the medicine because of the pain that cannot be tolerated and seek surgical treatment (5, 6).

In recent years, with the continuous development of imaging technology and nerve intervention, surgical treatment has gradually developed to be more mature. In 1983, Mullan's team first used percutaneous puncture to treat diseases, and achieved certain results. This laid the foundation for the germination of percutaneous balloon compression (PBC) in our country (7). The principle of PBC is to selectively damage the myelinated coarse fibers that conduct tactile sensation through balloon compression, while retaining the unmyelinated fine fibers that conduct pain, which is different from the known thermocoagulation radiofrequency because thermocoagulation radiofrequency can cause selective injury. It can be used as a useful supplement of microvascular decompression (MVD) for trigeminal neuralgia (8). PBC is not easy to cause corneal fiber injury. On the one hand, it may reduce the afferent sensation impulse and turn off the trigger switch of trigeminal nerve pain conduction pathway. On the other hand, it also relieves the nerve entrapment that may exist in the semilunar segment of the trigeminal nerve, so it has obvious advantages in the treatment of the I and II branches of trigeminal neuralgia (9).

Based on this, we observed 13 patients with primary trigeminal neuralgia in our hospital from April 2020 to July 2021, and discussed the therapeutic effect of PBC. The report was as follows.

## Materials and Methods

### Case Data

From April 2020 to July 2021, 13 patients with primary trigeminal neuralgia were treated in our hospital. Diagnosis criteria (10): (a) Pain history ≥3 months, and sudden and recurrent pain; There are no clinical symptoms during pain relief; (b) Pain distribution and one or more branches of the trigeminal nerve distribution region; The nature of pain is “cutting” or “electric shock;” Having a definite trigger point; (c) No positive signs on nervous system examination; (d) The attack form is fixed and rigid. Thirteen patients including eight females and five males. The average age of the patients was 71 years, left side pain in seven cases, right side pain in six cases, and the history of pain was 1–23 years, with an average of 5.3 years. All patients had received carbamazepine, oxcarbazepine, and other drug treatment, but with the prolongation of the course of disease, the patients gradually tolerated or could not tolerate the toxic and side effects of drugs, and all the patients were willing to undergo surgery. Two cases had undergone PBC and four cases had undergone microvascular decompression. Two patients underwent radiofrequency, PBC and MVD operations. The follow-up time ranged from 3 months to 1 year, with an average of 5 months. Before operation, all patients were routinely examined by MRI + trigeminal neurovascular imaging and 64-slice CT skull reconstruction to check the location and direction of foramen ovale. Exclusion criteria: Secondary trigeminal neuralgia caused by intracranial tumor compression; Complicated with intracranial aneurysm, cerebral and craniofacial vascular diseases or malformations, hydrocephalus and other neurological related diseases; Unable to tolerate surgery; Bilateral trigeminal neuralgia. Before operation, patients were informed of other alternative treatment schemes and asked to sign.

### Methods

#### Preoperative Preparation

**Figure 1:**
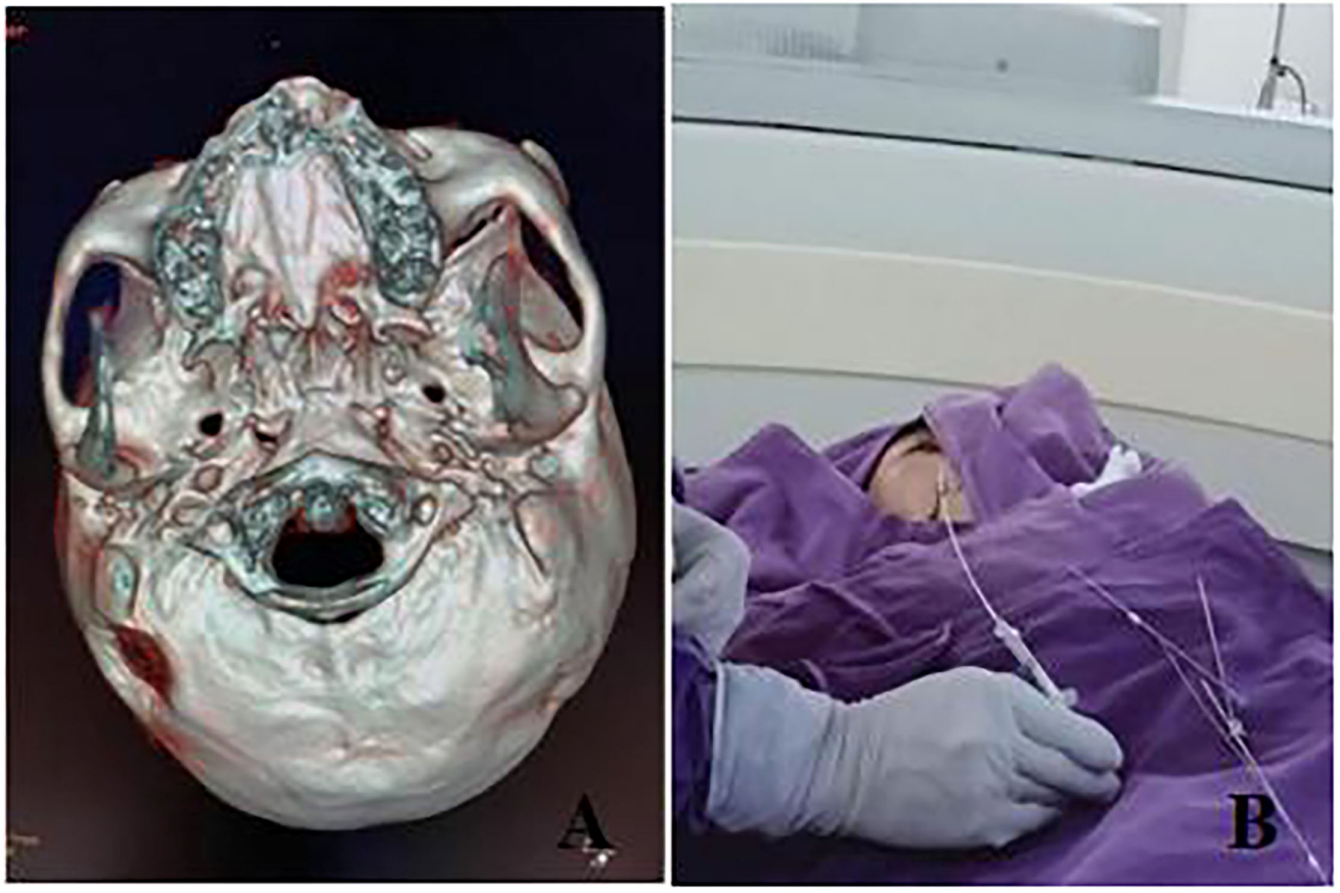
**(A)** 64-slice CT skull reconstruction was performed before surgery to check the direction and size of the foramen ovale; **(B)** Determine the direction of the patient's puncture.

**Figure 2:**
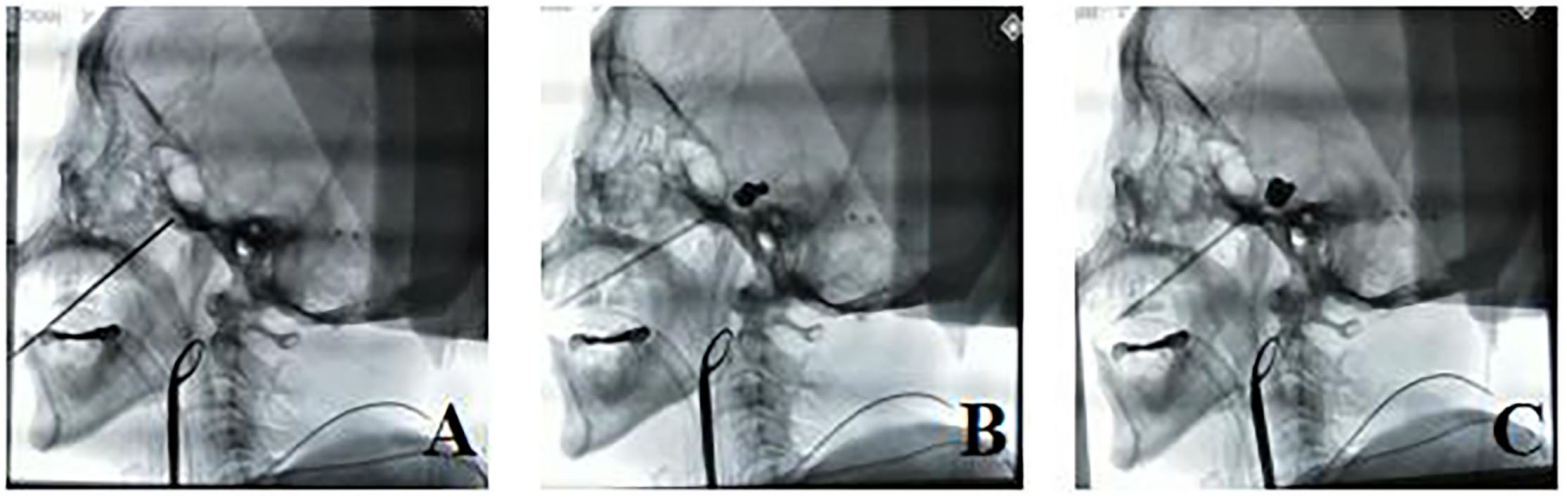
**(A–C)** After the puncture was in place during the operation, the balloon was filled, and the puncture point had to be inside Meckel's cave and formed an effective “pear” shape.

**Figure 3:**
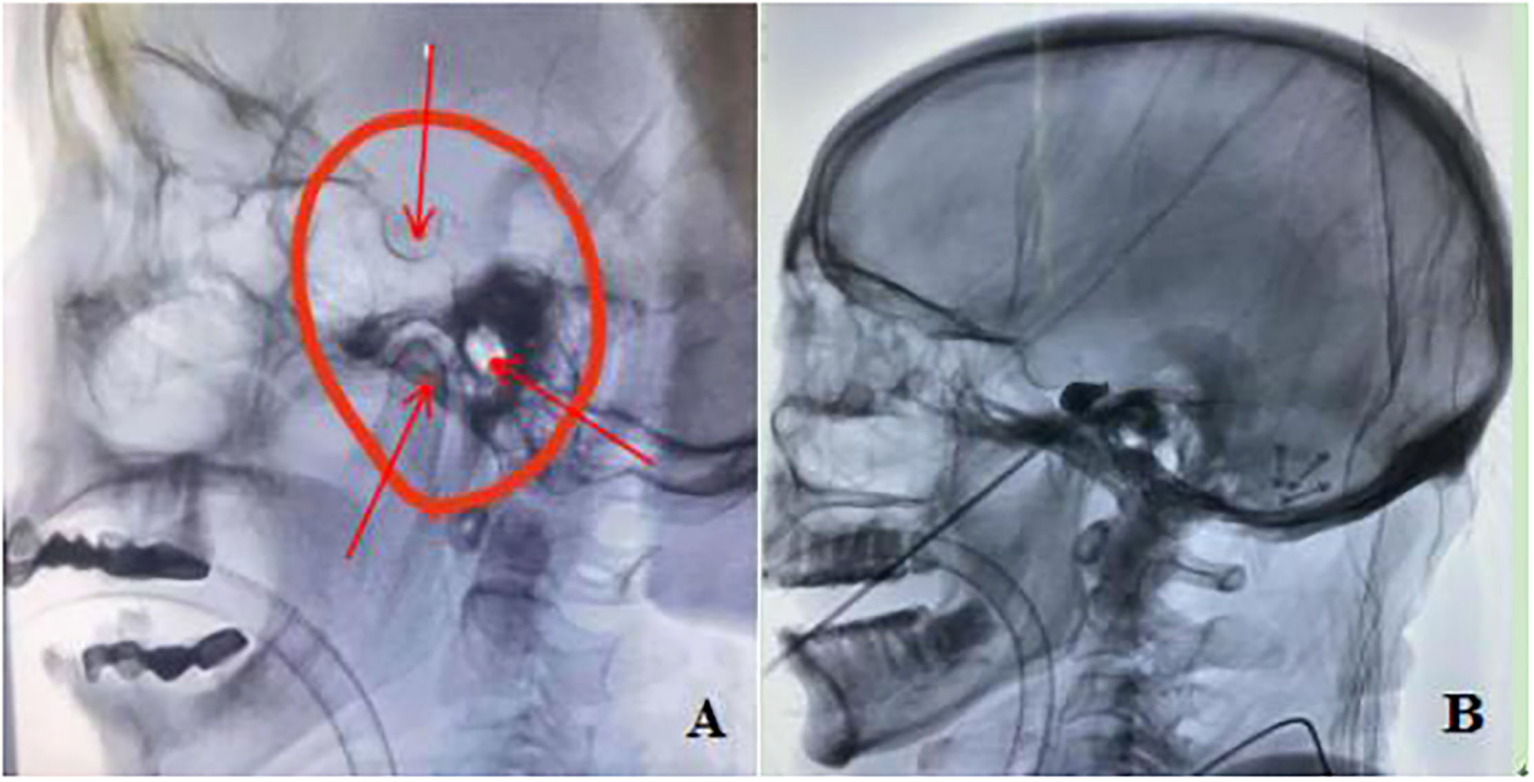
**(A)** When the posterior margin of the upper palate, temporomandibular joint and external auditory canal overlap (shown by arrow), it was considered a standard lateral position; **(B)** After the puncture needle pierces the dura mater of foramen ovale, it needs to be kept outside the skull to avoid further penetration, and the balloon will form an “inverted pear” shape after filling.

**Figure 4:**
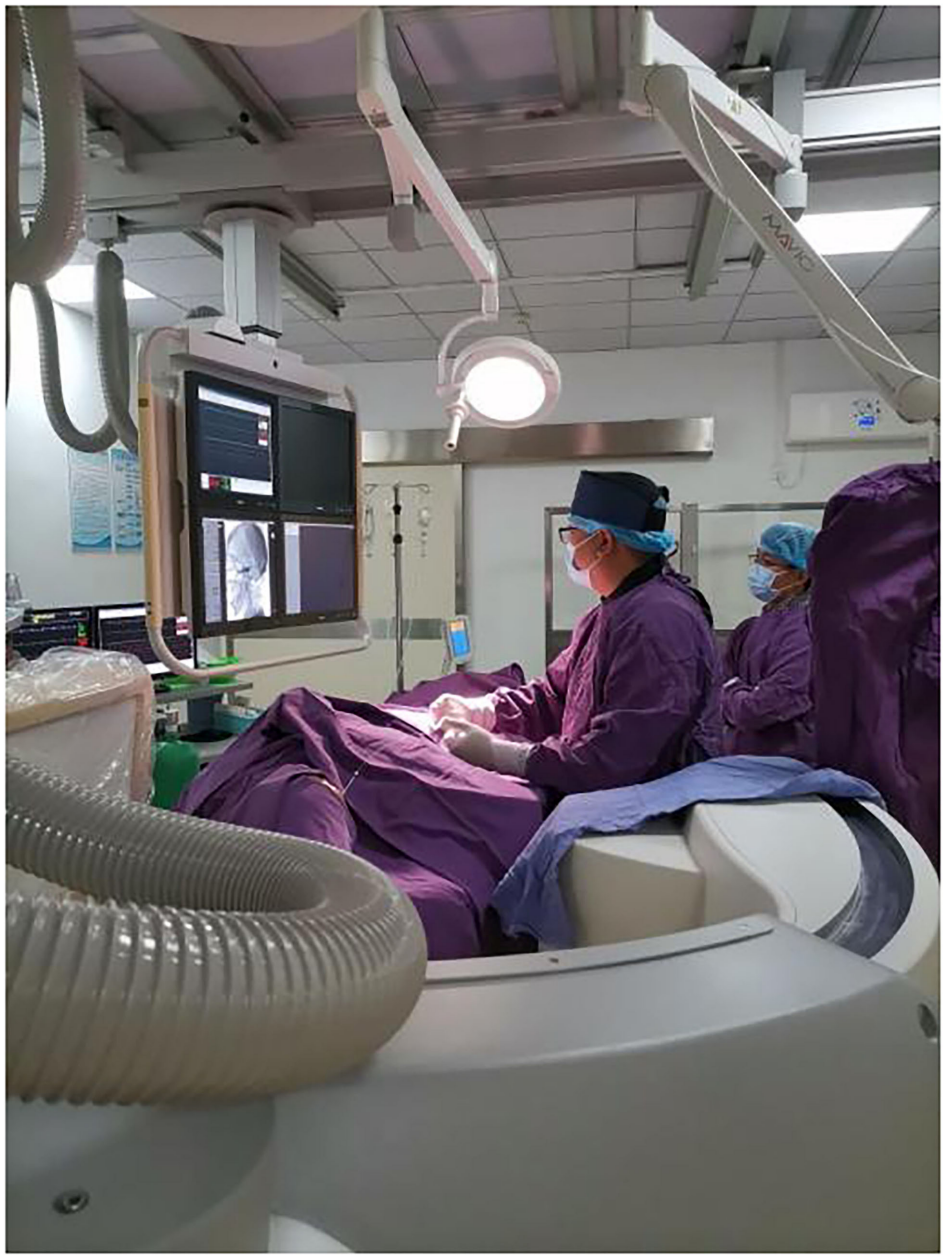
The puncture process was guided under the direction of DSA.

Trigeminal cardiac inhibitory reflexes have often occurred during PBC procedures, manifesting as transient but pronounced bradycardia during balloon filling. Before foramen ovale puncture, giving patients an appropriate amount of atropine and remifentanil could alleviate this reaction. Instructed patients to take supine position, and performed routine preoperative disinfection and towel spreading. Heart rate, blood pressure, and blood oxygen saturation were monitored in the whole process, and ambulatory arterial blood pressure was monitored in real time. Tracheal intubation, general anesthesia and breathing control, neck slightly extended, with the patient's nose as the highest point, and semilunar ganglion puncture surgery were carried out under the instructions of cross-line screen. (1) 64-slice CT skull reconstruction was performed before surgery, Hartel's anterior approach to the semilunar ganglion of trigeminal nerve was used, and the foramen ovale was the puncture target (Figure 1). The M-shaped puncture needle with a needle core was used as the puncture tool. The puncture point was 2.5–3 cm outside the mouth angle of the affected side, which basically corresponds to the root of the first molar. The other two reference points were the direction of the pupil on the same side, the outer canthus of the corner of the eye on the same side was connected to the external auditory canal, and the connection was 3 cm above the external auditory canal. Took the intersection of the two as the direction of the foramen ovale. (2) The needle was inserted from the puncture point, and the needle should be carefully inserted, it should be careful not to pierce the oral mucosa, and reached the foramen ovale under the instructions of the fluorescent screen, but to avoid penetrating the foramen ovale. The needle core was pulled out, and the blunt end of the 0.5 Kirschner wire was inserted into the catheter at the center of the introduction. The Kirschner wire and cannula were measured and fixed before the operation, so that the head end of the needle exceeds the puncture needle tip by 1–2 cm forward and enters into the semilunar segment's Meckel's cave (Figure 2). Until the needle feels obvious resistance, the needle core was pulled out. When the posterior margin of the upper palate, temporomandibular joint, and external auditory canal overlap, it was considered a standard lateral position, and the balloon was filled with the contrast agent iohexol under standard lateral DSA fluoroscopy, until the protrusion toward the posterior cranial fossa appeared “pear” shape (Figures 3, 4). If the puncture was not ideal, the cannula direction needed to be adjusted in time to re-puncture. The filling fluid of the balloon was 0.45–0.75 ml. It took 120–180 s to compress the semilunar segment, the contrast agent in the balloon was discharged, the catheter was pulled out, and the compression at the puncture point was about 5 min. Patients were taken into ICU for postoperative observation, and returned to ward after waking up.

### Evaluation Methods

VAS, VRS-4, and PPI were used to evaluate the postoperative pain relief. Complete pain relief: VAS < 10 points, VRS-4 0 grade or PPI 0 grade; Pain relief: VAS 10-40 points, VRS-4 1 grade, or PPI 1-2 grade: pain relief unsatisfactory: VAS ≥ 40 points, VRS-4 ≥ 2 grade, or PPI ≥ 3 grade. Different postoperative complications were analyzed, including facial numbness, masseter muscle weakness, herpes around the mouth, diplopia and so on.

## Results

All patients had a smooth operation, the inflation volume of the balloon was 0.7 ml, the average compression time was 120 s, and there was no balloon rupture during the operation. On the day after operation, 12 patients (92.3%) had complete pain relief, and 1 patient (7.7%) was not satisfied with pain relief, but the pain disappeared 2 weeks after the operation. After operation, there were 12 patients with facial numbness in the affected side (92.3%), three patients with masseter muscle weakness (23.0%), one patient with herpes around the mouth (7.6%), and one patient with diplopia (7.6%). Follow-up was conducted 3 months after operation, among which 13 cases were effectively followed up, and 1 patient had delayed effect due to the gradual reduction of oxcarbazepine after operation. The pain of this patient improved 1 month after operation, and the total effective rate was 92.3%. Twelve patients (92.3%) had slight numbness in the side of operation, and one patient had severe numbness in the side of operation with ulcer of the affected side, which was photophobia, which had certain influence on the quality of life of patients.

## Discussion

At present, the surgical treatment of trigeminal neuralgia can be roughly divided into four categories: MVD, stereotactic gamma knife, PBC, and radiofrequency thermocoagulation with glycerol injection (11). After MVD, the complete pain relief rate can reach 90%, and after 10 years, the complete relief rate can be maintained at about 70%, and the incidence of surgical complications is low, which is the most ideal surgical scheme at present (12). However, this operation is complicated, which requires high technical requirements for the operator, and is not suitable for patients who are weak, old, or have serious systemic diseases and cannot tolerate craniotomy under general anesthesia. Stereotactic gamma knife, as a non-invasive treatment method, was first applied in 1953. Although the long-term follow-up results show that gamma knife has a certain curative effect on trigeminal neuralgia, there are a large number of literature reports that the early postoperative complete pain relief rate is quite different (21.8–80%), and the rate of complete pain relief 10 years later was lower (45.3%), and the related surgical complications (such as facial sensory disturbance and numbness) and the recurrence rate of pain are also high (13). The rate of postoperative pain relief in PBC was 88.9–97.3%, and the rate of complete pain relief was about 62% in more than 10 years of follow-up. PBC also has a good curative effect on recurrent trigeminal neuralgia after microvascular decompression, the complete pain relief rate reached 82.7% in 3 years after operation (14). The effective relief rate of postoperative pain in this study was 92.3%, which was similar to previous reports and equivalent to the therapeutic effect of MVD. Compared with other surgical methods, PBC has four advantages. First, the wound is small, and the puncture needle is inserted beside the patient's mouth, only the pinhole size, less bleeding, less patient injury, less side effects, and fewer complications, etc. Second, the operation time is short, and the whole operation process can be completed in about 30 min. Third, the hospitalization time is short, and the patient can be discharged from hospital within 2–3 days after operation. Fourthly, the patient is well-tolerated, accepts the whole operation under general anesthesia, and the patient has less pain and discomfort during the operation (15–19).

Early balloon compression surgery lasts for a long time. Although the pain relief rate is high and the effective duration is long, facial sensory disturbance and myasthenia gravis tend to be severe and last for a long time, and there are many postoperative complications, so it is not recommended that the compression time be too long (20). The shorter the compression time, the higher the recurrence rate. Chen's team concluded that the appropriate compression time was 90 s, which not only ensured a high pain relief rate, but also did not increase the incidence of complications (21). In this study, the average time of compression was 120 s, range from 30 to 180 s. The filling volume of the balloon determines the pressure damage suffered by the semilunar ganglion nerve, which not only directly affects the pain relief effect after operation, but also leads to decreased facial sensation, numbness, and weakened masseter muscles. Clinical research has shown that surgery can't achieve the expected therapeutic effect when the balloon filling pressure was lower than 600 mmHg 0.45 ml, and surgery is suitable when the balloon pressure was 750–1,250 mmHg 0.65 ml (22). However, the pressure of each part of the balloon is not uniform, and the pressure at the semilunar node is the highest, which is significantly higher than that at the foramen ovale and the distal end. At present, few hospitals in China are equipped with pressure monitoring devices during surgery, and the general experience of surgeons is to inject 0.5–0.8 ml of non-ionic contrast agent. The average contrast agent injected in this group was 0.702 ml. Early balloon compression surgery will continue to have numbness at 1 year after operation, which can't be alleviated. Patients who experienced severe numbness after early balloon compression surgery, none of the patients improved, and the masseter muscle was atrophied and facial changes appeared afterwards (23, 24). In this study, six patients with severe numbness on the affected side of the face had an ideal “inverted pear” shaped balloon filling shape during the operation, the balloon filling volume was 0.7 ml, and the balloon filling time was 20–25 min. Compared with other patients, there was no special situation, it was speculated that the serious numbness on the affected side of the face was caused by anatomical factors: the Meckel's cave volume of the patient was too small, although the balloon was filled with the same volume of contrast agent, the pressure generated in the semilunar ganglion was obviously high, resulting in extensive destruction of nerve fibers. Patients with PBC with a long duration of intraoperative compression often have severe postoperative facial numbness, but the relative relief of postoperative facial pain is more obvious. Therefore, clinically, it is necessary to appropriately extend or shorten the compression time according to the severity of preoperative pain, but it should be within a reasonable range.

## Conclusion

PBC is a minimally invasive, safe, effective, and minimally invasive treatment with minor complications. The indications of PBC for trigeminal neuralgia are: (1) Those who are afraid of craniotomy and refuse craniotomy; (2) Elderly and infirm people with more basic diseases; (3) Patients with poor general health; (4) Patients who have side effects on drugs; (5) Patients with ineffective microvascular decompression or postoperative pain recurrence; (6) Patients with secondary trigeminal neuralgia who have poor craniotomy treatment effect or can't tolerate drug (25, 26). Compared with other surgical methods, the biggest advantage of PBC is that it is easy to master, which is conducive to the popularization and development of primary hospitals. However, we should also realize that even if the balloon filling volume and filling compression time are reasonable, a small number of patients will still be accompanied by severe facial numbness and muscular atrophy after operation, which will affect the quality of life to a certain extent. How to reduce postoperative complications is the focus of our attention in the future, and whether there is recurrence or not in the 3 and 5 year follow-up period after surgery is also the focus of our attention in the future. Comparative analysis of a large number of cases with different surgical methods is the most effective means to evaluate the surgical efficacy.

## Data Availability Statement

The original contributions presented in the study are included in the article/supplementary material, further inquiries can be directed to the corresponding author.

## Ethics Statement

The studies involving human participants were reviewed and approved by the Medical Ethics Committee of Pingkuang General Hospital. The patients/participants provided their written informed consent to participate in this study. Written informed consent was obtained from the individual(s) for the publication of any potentially identifiable images or data included in this article.

## Author Contributions

HW was the instructor of the study. CC and DC were responsible for the design of the study. FL and SH were responsible for collecting clinical data. WD and JW were responsible for data evaluation and recording. WC was responsible for data statistical analysis and papers of writing. All authors contributed to the article and approved the submitted version.

## Conflict of Interest

The authors declare that the research was conducted in the absence of any commercial or financial relationships that could be construed as a potential conflict of interest.

## Publisher's Note

All claims expressed in this article are solely those of the authors and do not necessarily represent those of their affiliated organizations, or those of the publisher, the editors and the reviewers. Any product that may be evaluated in this article, or claim that may be made by its manufacturer, is not guaranteed or endorsed by the publisher.
